# Comparing dynamic causal models of neurovascular coupling with fMRI and EEG/MEG

**DOI:** 10.1016/j.neuroimage.2020.116734

**Published:** 2020-08-01

**Authors:** Amirhossein Jafarian, Vladimir Litvak, Hayriye Cagnan, Karl J. Friston, Peter Zeidman

**Affiliations:** a The Wellcome Centre for Human Neuroimaging, University College London, UK; b MRC Brain Network Dynamics Unit (BNDU) at the University of Oxford, Oxford, UK; c Nuffield Department of Clinical Neurosciences, University of Oxford, Oxford, UK

**Keywords:** Dynamic causal modelling, Multimodal, Neurovascular coupling, Neural mass models, Bayesian model comparison

## Abstract

This technical note presents a dynamic causal modelling (DCM) procedure for evaluating different models of neurovascular coupling in the human brain – using combined electromagnetic (M/EEG) and functional magnetic resonance imaging (fMRI) data. This procedure compares the evidence for biologically informed models of neurovascular coupling using Bayesian model comparison. First, fMRI data are used to localise regionally specific neuronal responses. The coordinates of these responses are then used as the location priors in a DCM of electrophysiological responses elicited by the same paradigm. The ensuing estimates of model parameters are then used to generate neuronal drive functions, which model pre- or post-synaptic activity for each experimental condition. These functions form the input to a model of neurovascular coupling, whose parameters are estimated from the fMRI data. Crucially, this enables one to evaluate different models of neurovascular coupling, using Bayesian model comparison – asking, for example, whether instantaneous or delayed, pre- or post-synaptic signals mediate haemodynamic responses. We provide an illustrative application of the procedure using a single-subject auditory fMRI and MEG dataset. The code and exemplar data accompanying this technical note are available through the statistical parametric mapping (SPM) software.

## Highlights

• A method is introduced for Bayesian fusion of M/EEG and BOLD fMRI data using dynamic causal modelling.
• The key novel contribution is the introduction of neural drive functions, which link models of the two modalities.
• Bayesian model comparison is used to select plausible hypotheses about the function of neurovascular coupling.

## 1 Introduction

To interpret the blood oxygenation-level dependent (BOLD) contrast, and its disruption due to aging (Tarantini et al., 2017), disease (Shabir et al., 2018), or pharmacological interventions (Otsu et al., 2015), a better understanding of the biological mechanisms of neurovascular coupling would be useful. Neuronal activity triggers vasodilation, both directly via signalling molecules – such as nitric oxide and adenosine (Li and Iadecola, 1994) – and indirectly via astrocytes (Takano et al., 2006). The ensuing change in blood flow is accompanied by a change in blood oxygenation (Logothetis et al., 2001; Filosa and Blanco, 2007), detectable as the BOLD contrast. However, there are many outstanding questions about the origin of BOLD in the human brain (Arthurs and Boniface, 2002; Hall et al., 2016). For instance, is it driven by pre- or post-synaptic potentials of neuronal populations? Does a region’s BOLD response depend on local or distal neuronal projections? What causes region-specific differences in the BOLD response? Human neuroimaging can complement animal models in addressing these fundamental questions. Moreover, neuroimaging is uniquely placed for investigating differences between people with different aetiologies or at different stages of disease progression, non-invasively. Aberrant neurovascular coupling may play a role in many neuro-physiological conditions (Tarantini et al., 2017). For instance, in Alzheimer’s disease, a reduction in induced blood flow – in response to neuronal demands for energy – has been implicated in cognitive decline (Shabir et al., 2018; Snyder et al., 2015). Another example is aging, where there is a progressive reduction in the efficacy of neurovascular coupling (Lipecz et al., 2019). These motivate the importance of an efficient approach to disambiguate the neurovascular mechanisms that underwrite neural and haemodynamic responses.

Invasive recordings in animal models are commonly employed to distinguish neuronal, vascular and haemodynamic contributions to the BOLD response (e.g. Logothetis et al., 2001; Grill-Spector et al., 2006; Shmuel et al., 2006). However, the same imaging techniques cannot be adopted to study the human brain in vivo, which necessitates the use of non-invasive functional imaging. BOLD contrast imaging using fMRI provides high spatial resolution for localising activity and, with suitable models, enables inferences about the mechanisms of neurovascular coupling (Stephan et al., 2007). This imaging technique typically has greater temporal resolution than other MRI methods used to study neurovascular coupling, such as arterial spin labelling (Ferré et al., 2013) or Vascular-Space-Occupancy (Lu and van Zijl, 2012); however, it is still too slow to inform detailed models of neuronal activity. By contrast, electromagnetic recordings such as MEG provide exquisite temporal resolution – at the level of electrophysiological dynamics – which in turn support the identification of detailed neural models (David et al., 2006). The question then arises: how can we leverage the sensitivity of fMRI to haemodynamics and the sensitivity of MEG to neuronal dynamics to best study neuro-vascular interactions in humans non-invasively? The approach pursued here is to combine a detailed neuronal model fitted to EEG or MEG data with a model of neurovascular coupling and haemodynamics fitted to fMRI data. Our objective in this paper is to introduce efficient tools useful for modelling neurovascular function, rather than providing answers to long standing questions about the origin of the BOLD signal. Therefore, at this stage, we do not intend to draw any definitive conclusions about neurovascular physiology (which will require group studies). Instead, we present the methodological foundation by which competing hypotheses about the origin of the BOLD response can formulated and tested. We envisage this method will be particularly useful for modelling between-subject differences in neurovascular coupling due to pathology and disease. To illustrate how to apply the methods, we use an empirical dataset, in which a single subject performed an auditory (roving oddball) task, while undergoing MEG and fMRI.

To establish a method for modelling neurovascular coupling, our first consideration was which neuronal model to use. Neuronal models of varying complexity have been used in previous studies examining neurovascular coupling. For example, Riera et al. (2005, 2006, 2007) explored mechanisms of neurovascular coupling using fMRI- EEG data. In their models, the BOLD response could be induced by pre- and/or post-synaptic potentials associated with a single population of deep pyramidal cells, connected with two populations of inhibitory interneurons. Voges et al. (2012) investigated neurovascular coupling in the context of epilepsy, using a neural mass model with one inhibitory and one excitatory sub-population, based on Wendling et al. (2000, 2005) and Jansen and Rit (1995). A recent study by Friston et al. (2017) used a four population canonical microcircuit (CMC) model (Bastos et al., 2012) to demonstrate that fMRI and EEG/LFP data features may be uncorrelated, despite having the same underlying neuronal sources. They coupled the CMC model, which includes superficial and deep pyramidal cells as well as excitatory and inhibitory neurons, with the haemodynamic model typically employed in DCM for fMRI (Stephan et al., 2007). This combined model, which so far has only been demonstrated with simulated EEG/LFP data, has the potential to reveal laminar specific contributions to the BOLD response. For this reason, we used the CMC model here, although it could easily be replaced with any other appropriate neural mass model.

Our second consideration was the form of the neurovascular coupling model and which neuronal sources should drive haemodynamics. Previous studies have explored detailed neurovascular coupling models using non-invasive measurements (see review by Huneau et al., 2015). For example, Sotero and Trujillo-Barreto (2007) proposed a model in which lumped excitatory and inhibitory neuronal inputs drive a detailed model of metabolic change and haemodynamics. Other models have been evaluated by Rosa et al. (2011), who embedded the forward model proposed by Riera et al. (2006) in a (variational) Bayesian framework. They performed a Bayesian model comparison to evaluate different neuro-vascular coupling functions based on synaptic activity and/or post-synaptic firing rates. Here, we took a similar approach and compared the evidence for different combinations of pre- or post-synaptic neuronal inputs, as well as exogenous inputs from different neuronal populations, using Bayesian model comparison. These mixtures of neuronal activity entered an established neurovascular coupling model (Friston et al., 2000) in which a vasodilatory signal induces flow and is subject to feedback induced by that flow. This lumped vasoactive signal is likely to subsume various signalling molecules and cascades. Nitric oxide (NO) was proposed as a likely basis for this signal, as its half-life is consistent with empirically derived parameter estimates from fMRI measurements (Friston et al., 2000). Nevertheless, there are many other vasoactive agents that constrict or dilate blood vessels, including epoxyeicosatrienoic acids (EETs), prostaglandin E2 (PGE_2_) and potassium (K^+^). (For a recent review, see Nippert et al., 2018). If distinguishing these signalling pathways is of interest, more biophysically detailed models could be implemented using the model comparison framework presented below (e.g., see Huneau et al., 2015). In the illustrative model used here, the lumped vasoactive signal drives a haemodynamics model, and a subsequent model of the fMRI signal (Stephan et al., 2007). We emphasise, that any of these components could be substituted or compared based on their contribution to model evidence.

Our third consideration was how to integrate MEG and fMRI data to efficiently estimate the parameters of the neuronal, neurovascular and haemodynamic parts of the model. To make inversion tractable, reasonable independence assumptions can be made about the parameters (i.e. a mean-field approximation). For example, Rosa et al. (2011) used a three-step variational Bayesian estimation procedure, where they first estimated neuronal parameters, then neurovascular coupling parameters, and finally the parameters governing haemodynamics. Here, we also used variational Bayesian inference methods, and divided the estimation into a neuronal part and a neurovascular and haemodynamics part, linked by *neuronal drive functions*. These functions are canonical synaptic responses to each experimental condition from each neuronal population, derived from a neural mass model which has been fitted to the MEG data. These functions then form the input to the neurovascular coupling model, which in turn drives the haemodynamics. Parameters relating to the neurovascular and haemodynamic parts of the model are estimated from the fMRI data. This approach offers convenience and flexibility, because the neuronal drive functions can be generated from any of the neural mass models available in the DCM framework without the need for re-implementation.

In summary, the framework we set out in this paper couples a dynamic causal model of laminar specific neuronal responses (Bastos et al., 2012, 2015) with a model of neurovascular coupling and the BOLD response (Stephan et al., 2007). They are linked by neuronal drive functions, which model the pre- or post-synaptic activity of each neuronal population under each experimental condition. The form of the neuronal drive or coupling functions is parameterised to enable hypothesis testing using Bayesian model comparison. To illustrate the proposed approach in this paper, we specified a sample factorial model space covering a number of foundational questions about the mechanisms of neurovascular coupling. The factors were: presynaptic versus postsynaptic contributions to the neurovascular signal, whether the inputs to neurovascular coupling were region-specific, whether distal regions contributed to local changes in BOLD response, and whether neurovascular delays associated with the release of vasoactive agents (e.g. calcium) should be modelled. This model space allowed us to illustrate how to perform family-wise model comparisons, quantifying the evidence for each question in turn. Future studies may use a model space such as this to examine the commonalities or differences among individuals or groups of subjects in their neurovascular coupling.

This paper has five sections. In section two, we set out the theory underlying the approach. In section three, to further unpack intricacies of the methodology, we illustrate multimodal DCM applied to an exemplar fMRI/MEG dataset and compare models associated with some key hypothesis about neurovascular mechanisms. The Discussion, in section four, considers the procedures in terms of limitations and future applications. Finally, in section five, a software note provides instruction on the code, as implemented in a toolbox for SPM.

## 2 Theory

### 2.1 Dynamic causal modelling for MEG

**Fig. 1:**
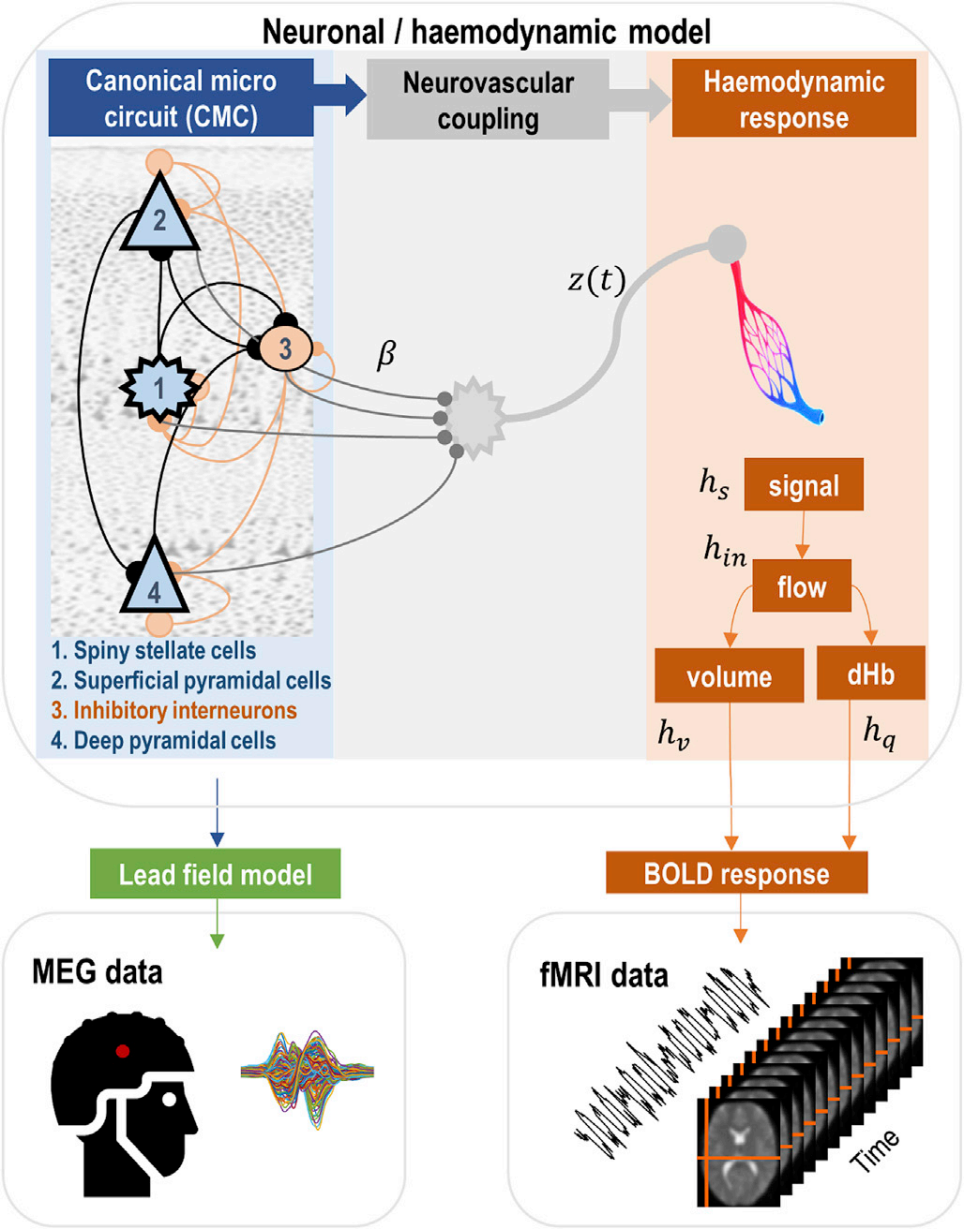
Components of a forward model of fMRI and electrophysiological (MEG) data. The generative neuronal/haemodynamic model is shown in the top panel, which illustrates the pathway from neural populations (blue panel on the left) to neurovascular coupling (grey panel in the centre) and haemodynamic response (orange panel on the right). The neural model (left panel) is a laminar specific canonical microcircuit (CMC) comprising four populations (numbered 1–4) per brain region. Each CMC is linked through extrinsic (between regions) forward and backward connections. Pre- or postsynaptic neuronal signals β are combined (at the level of the putative astrocytes) which is presented in the middle panel. The ensuing neurovascular signal z(t) at time t drives the haemodynamic part of the model (right panel). This accounts for increased blood flow to the venous compartment (pictured) and is accompanied by changes in blood volume and the level of deoxyhaemoglobin. The bottom panels illustrate the electrophysiological and fMRI measurements that arise from the neuronal and haemodynamic parts of the model respectively, mediated by a spatial lead field model for MEG and a BOLD signal model for fMRI. To make inversion of this model tractable, we split the neuronal and haemodynamic parts and connected them via neuronal drive functions – see text and Fig. 5.

A biologically informed generative model of multimodal fMRI and MEG data is shown in Fig. 1. This DCM includes the common underlying neuronal generators of both MEG and fMRI measurements, mediated by a spatial lead field and BOLD response model, respectively. We will explain each part of the model in the following sections, before illustrating its application to real data. All variables are defined in Table 1, Table 2, Table 3, Table 4.

**Table 1:** Parameters of the neuronal model (see also Fig. 1).

	Description	Parameterisation	Prior
$\kappa_{i}$	Postsynaptic rate constant of the $i^{th}$ neuronal population in each of *N* regions	$\begin{array}{l} \exp\left(\theta_{\kappa }\right)\cdot \kappa_{i}\\ \kappa =\left[256,128,16,32\right] \end{array}$	$p\left(\theta_{\kappa }\right)=N\left(0,0\right)$
$a_{i\to k}$	Intrinsic connectivity between populations *i* and *k* in each region.	$\exp\left(\theta_{a}\right)\cdot a$ a=[2111]∗512	$p\left(\theta_{a}\right)=N\left(0,0\right)$
$B_{b,f}$	Condition-specific matrices. Elements are zero unless forward, backward or intrinsic connections are allowed to change in different conditions.	$\theta_{b,f}$	$p\left(\theta_{b}\right)=N\left(0,\frac{1}{8}\right)$
$A_{f,b}$	Forward and backward extrinsic connectivity matrices. If there is any forward (backward) connection between from region j to i, the corresponding element (i,j) in $A_{f}$ (respectively $A_{b})$ is set to one.	$\exp\left(\theta_{A}\right).A_{f,b\;}$	$p\left(\theta_{A}\right)=N\left(0,\frac{1}{8}\right)$
C	Scalar matrix to driving input	$\theta_{c}$	$p\left(\theta_{C}\right)=N\left(0,\frac{1}{32}\right)$

**Table 2:** Parameters of neurovascular and haemodynamic response functions.

	Description	Parameterisation	Prior
η	Rate of signal decay per sec	$0.64\cdot \exp\left(\theta_{\eta }\right)$	$p\left(\theta_{\eta }\right)=N\left(0,\frac{1}{256}\right)$
χ	Rate of flow-dependent elimination	$0.32\cdot \exp\left(\theta_{\chi }\right)$	$p\left(\theta_{\chi }\right)=N\left(0,0\right)$
τ	Rate of hemodynamic transit per sec	$2.00\cdot \exp\left(\theta_{\tau }\right)$	$p\left(\theta_{\tau }\right)=N\left(0,\frac{1}{256}\right)$
α	Grubb’s exponent	$0.32\cdot \exp\left(\theta_{\alpha }\right)$	$p\left(\theta_{\alpha }\right)=N\left(0,0\right)$
ε	Intravascular: extravascular ratio	$1.00\cdot \exp\left(\theta_{\varepsilon }\right)$	$p\left(\theta_{\varepsilon }\right)=N\left(0,\frac{1}{256}\right)$
ϕ	Resting oxygen extraction fraction	$0.40\cdot \exp\left(\theta_{\phi }\right)$	$p\left(\theta_{\phi }\right)=N\left(0,0\right)$
$\beta_{i}$	Sensitivity of signal to neural activity	$\theta_{\beta }$	$p\left(\theta_{i}\right)=N\left(0,\frac{1}{16}\right)$
$\tau_{nc}$	Decay rate of the astrocytes collateral	$0.7\cdot \text{exp}\left(\theta_{\tau_{nc}}\right)$	$p\left(\theta_{nc}\right)=N\left(0,\frac{1}{16}\right)$

**Table 3:** Biophysical parameters of the BOLD observation model in equation (10).

	Description	Value
$V_{0}$	Blood volume fraction	0.08
$k_{1}$	Intravascular coefficient	6.9⋅ϕ
$k_{2}$	Concentration coefficient	ε⋅ϕ
$k_{3}$	Extravascular coefficient	1−ε

**Table 4:** Glossary of variables and expressions.

Variable	Description
u	Thalamic input, modelled by a Gaussian function.
$V_{j}^{K}$	The *j*-th (neuronal) state in region *K*; e.g., mean depolarisation of a neuronal population
$\sigma \left(V_{j}^{K}\right)$	The neuronal firing rate – a sigmoid squashing function of depolarisation
z	Neurovascular signal; e.g., intracellular astrocyte calcium levels
$h_{s},h_{in},h_{v}\;,h_{q}$	Haemodynamic states: h_s_ - vasodilatory signal (e.g., NO), h_in_ - blood flow, h_v_ - blood –volume and h_q_ - deoxyhaemoglobin content
$\Psi_{j}$	Electromagnetic field vector mapping from (neuronal) states to measured (electrophysiological) responses

#### 2.1.1 Generative model of neuronal responses

We used the canonical microcircuit (CMC), which models the circuitry of a typical cortical column (Bastos et al., 2012, 2015; Douglas and Martin, 1991). The model has been widely applied in the translational neuroscience literature, in particular in the context of predictive coding (Bastos et al., 2012), to explain evoked (Rosch et al., 2019) and spectral responses (Rosch et al., 2018). It comprises four neuronal populations per brain region: spiny stellate cells in the granular layer (ss), superficial pyramidal cells in the supragranular layer (sp), inhibitory interneurons distributed in all layers of the cortex (ii) and deep pyramidal cells in the infragranular layers (dp), as shown in Fig. 1. The connectivity architecture in the CMC model introduced here consists of a subset of known anatomical connections (predominantly) in visual hierarchies of cortex (Ninomiya et al., 2012; Friston et al., 2017). The four populations within each cortical column have intrinsic (inter-and intra-laminar) connections that are ubiquitous in most cortical areas (Thomson and Bannister, 2003; Binzegger et al., 2004; Haeusler and Maass, 2007). Experimental and extrinsic inputs are received by spiny stellate cells in the granular layer (hereinafter referred to as extrinsic forward connections) that project to superficial pyramidal cells and thereafter to deep pyramidal cells. Each excitatory connection establishes reciprocal connections with inhibitory interneurons. All populations have a recurrent (self) inhibitory connection proportional to the level of excitation of the neuronal population. There are two types of external (extrinsic) input entering each microcircuit from different levels of the cortical hierarchy. Inputs can be bottom-up (forward) connections arising from superficial pyramidal cells of the level below, targeting spiny stellate cells and deep pyramidal cells. Alternatively, inputs can be top-down (backward) connections arising from deep pyramidal cells of the level above, targeting inhibitory interneurons and superficial pyramidal cells (Felleman and Van Essen, 1991; Hilgetag et al., 2000).

Two conversion operators govern the dynamics of each neuronal population (Jansen and Rit, 1995). The first operator converts the mean pre-synaptic firing rate m to the mean postsynaptic membrane potential V as follows (Freeman, 1975):

(1) V=h⊗m

where ⊗ denotes the linear convolution operator and h is the impulse response function (synaptic kernel) with synaptic rate constant κ:

(2) $$h\left(t\right)=\{\begin{array}{c} \frac{t}{\kappa }e^{-\frac{t}{\text{ ​}\kappa }},t\geq 0\\ 0,t<0 \end{array}$$

The second operator then transforms the postsynaptic membrane potential into a firing rate, which forms the input to the next connected neural population:

(3) $$\sigma \left(V\right)=\frac{1}{1+\exp(-a_{s}\;\left(V-V_{th}\right))}-\frac{1}{1+\text{exp}\left(a_{s}V_{th}\right)}$$

In equation (3), $a_{s}$ is the slope of the sigmoid function (in DCM, it is set to one) and $V_{th}$ is the firing threshold (in DCM it is set to zero, see Moran et al., 2007). This effectively means neuronal firing is treated as a deviation from baseline firing; thereby allowing for negative firing rates (Moran et al, 2007, 2013 and Jirsa and Haken, 1997). This is fairly common for convolution type mass models of the sort used here e.g., Jirsa and Haken (1997). In addition, please see Moran et al. (2013) for a discussion of other kinds of models (e.g., conduction-based models) where nonnegative firing constraints are explicit. The maximum firing in equation (3) is set to one because – in this parameterisation – the maximum firing rate is lumped with the connectivity constants (e.g., Jirsa and Haken, 1997). The dynamics of postsynaptic potentials in region K, population i, $V_{i}^{K},$ with the synaptic rate constant $\kappa_{i}$ obey second order differential equations as follows:

(4) $${\left(1+\frac{1}{\kappa_{i}}\frac{d}{dt}\right)}^{2}V_{i}^{K}\left(t\right)\;=f_{i}\left(V_{ex}^{o},V_{i}^{K},u\right)$$

where the intrinsic presynaptic excitations are given by $V_{i}^{K}$, the term $V_{ex}^{o}$ denotes extrinsic drives of a population o in a distal region ex ; and the function f is defined as follows (Friston et al., 2017):

(5) $$f_{i}\left(V_{ex}^{\sigma },V_{i}^{K},u\right)=\;\{\begin{array}{c} A_{f}^{sp\to ss}\sigma \left(V_{sp}^{ex}\right)-a_{ss\to ss}\sigma \left(V_{ss}^{k}\right)-\;a_{sp\to ss}\sigma \left(V_{sp}^{k}\right)-a_{ii\to ss}\sigma \left(V_{ii}^{k}\right)+Cu_{k}\;\;\;\;\;\;\;\;\;\;\;\;\;\;\;\;\;\;\;if\;i=ss\\ A_{b}^{dp\to sp}\sigma \left(V_{dp}^{ex}\right)-a_{sp\to sp}\sigma \left(V_{sp}^{k}\right)+\;a_{ss\to sp}\sigma \left(V_{ss}^{k}\right)-a_{ii\to sp}\sigma \left(V_{ii}^{k}\right)\;\;\;\;\;\;\;\;\;\;\;\;\;\;\;\;\;\;\;\;\;\;\;\;\;\;\;\;\;\;if\;i=sp\\ A_{b}^{dp\to ii}\sigma \left(V_{dp}^{ex}\right)-a_{ii\to ii}\sigma \left(V_{ii}^{k}\right)-a_{dp\to ii}\sigma \left(V_{dp}^{k}\right)+\;a_{ss\to ii}\sigma \left(V_{ss}^{k}\right)+a_{sp\to ii}\sigma \left(V_{sp}^{k}\right)\;\;\;\;\;if\;i=ii\\ A_{f}^{sp\to dp}\sigma \left(V_{sp}^{ex}\right)-a_{dp\to dp}\sigma \left(V_{dp}^{k}\right)-\;a_{ii\to dp}\sigma \left(V_{ii}^{k}\right)+a_{sp\to dp}\sigma \left(V_{sp}^{k}\right)\;\;\;\;\;\;\;\;\;\;\;\;\;\;\;\;\;\;\;\;\;\;\;\;\;\;\;if\;i=dp \end{array}$$

The laminar specificity of the extrinsic and intrinsic connections in equation (5) are specified by placing prior constraints on the intrinsic (within-region) connectivity parameters $a_{*\to *}$ as well as on the elements of the extrinsic (between-region) forward and backward adjacency matrices $A_{f,b}^{*\to *}$ ($A_{f}^{sp\to ss}$ and $A_{f}^{sp\to dp}$ denote forward connections matrices, whereas backward connection matrices are specified by $A_{b}^{dp\to sp}$ and $A_{b}^{dp\to ii}$). Matrix C parameterises the experimental driving input entering the system. These modelled neuronal dynamics are the common source of both the fMRI and MEG signals. As we will explain later, in DCM for MEG, we estimate condition specific forward and backward matrices $B_{f,b}$, which are applied (algebraically added) to the $A_{f,b}$ matrices and $a_{*\to *}$ parameters in order to model the differences between experimental conditions.

#### 2.1.2 MEG observation model

The observation function for MEG data has the following form (Daunizeau et al., 2009):

(6) $$y_{MEG}=\sum_{K}{\text{Υ}}^{K}{\text{Δ}}_{0}^{K}\sum_{j}\Psi_{j}V^{j}\left(t\right)+\varepsilon_{M}$$

where $\varepsilon_{M}\sim N\left(0,C_{M}\right)$ are I.I.D. measurement errors (with covariance matrix $C_{M}$), ${\text{Υ}}^{K}$ is a gain matrix for brain region K and ${\text{Δ}}_{0}^{K}$ is a Laplacian operator that is modelled as a mixture of n spatial basis functions as follows:

(7) $${\text{Δ}}_{0}^{K}=\sum_{n}{\text{Λ}}_{n}^{K}{\text{Θ}}_{n}^{K}$$

where ${\text{Λ}}_{n}^{K}$ are the spatial eigenvalues of the gain matrix and ${\text{Θ}}_{n}^{K}$ are parameters to be estimated. The term $\sum_{j}\Psi_{j}V^{j}\left(t\right)$ (where j is the index of neuronal population) in equation (6) quantifies the contribution (modelled by unknown vector $\Psi_{j}$) of neuronal populations denoted by $V^{j}\left(t\right)$ to the MEG signal. This completes the forward model of MEG data.

### 2.2 Haemodynamic model

#### 2.2.1 Generative model of neurovascular coupling

Neuronal dynamics (presynaptic or postsynaptic) excite neurovascular coupling mechanisms, which in turn trigger the vascular system to provide oxygen for neuronal consumption. While detailed models of the neurovascular system have been developed (e.g. Carmignoto and Gomez-Gonzalo, 2010; Figley and Stroman, 2011), the lack of temporal resolution of fMRI places a limit on the complexity of models that can be inverted efficiently (Huneau et al., 2015; Pang et al., 2017). The framework set out in this paper provides the necessary tools for comparing the evidence for models of neurovascular coupling, enabling one to select the model(s) that optimise the trade-off between accuracy and complexity. Two groups of models will be compared in this paper to illustrate the approach.

The first group of models posit that an instantaneous neurovascular response to neuronal activity (presynaptic firing rates or postsynaptic potentials) gives rise to the BOLD response. This is mediated by the release of signalling molecules that regulate and induce blood flow. The neurovascular signal can therefore be characterised as the algebraically scaled and summed responses associated with different neuronal populations. The scaling can either be considered to be the same for all regions, or different across regions: we will compare the evidence for each of these options below. Additionally, we will compare models where presynaptic inputs to each of the neuronal populations in the CMC were grouped into inhibitory, excitatory and extrinsic signals, each scaled by global coefficients (equal across regions) and summed to generate inputs to the haemodynamic model, as proposed in Friston et al. (2017). Grouping the neuronal contributions in this way offers a more parsimonious model than parameterising every neuronal population’s contribution. Here, all scale values associated with the neurovascular parameters had a (relatively) flat prior, placing minimal constraints on their value.

Alternatively, there might be a delay between the neuronal activity and haemodynamic response, due to the kinetics of intracellular calcium levels in the collaterals of astrocytes (Bazargani and Attwell, 2016). Therefore, a second class of neurovascular models was included with additional delay factors. A parsimonious model that captures the mean delay with time constant $\tau_{nc}$ due to elevation of intracellular calcium level is governed by a second order linear system with an impulse response function proposed by Pang et al. (2017):

(8) $$f_{nc}\left(t\right)=\left\{\begin{array}{ll} \frac{t}{\tau_{nc}}e^{-\frac{t}{\tau_{nc}}}\;, & t\geq 0\\ 0, & t<0 \end{array}\right)$$

The prior expected value of the delay factor in equation (8) was 0.7s based on recent observations from animal studies (Masamoto et al., 2015).

#### 2.2.2 Generative model of the BOLD response

A linear transformation of the neurovascular coupling signal gives the vasodilatory signal that alters the blood flow and accordingly the blood volume and oxygenation level. The haemodynamic model explains the dynamics of the vascular system as follows (Friston et al., 2000, Friston et al., 2003):

(9) $$\begin{array}{cc} \dot{h_{s}} & =z-\eta h_{s}-\chi \left(h_{in}-1\right)\\ {\dot{h}}_{in} & =h_{s}\\ \dot{h_{v}} & =\frac{1}{\tau_{h}}\left(h_{in}-h_{v}^{\frac{1}{\alpha }}\right)\\ \dot{h_{q}} & =\frac{1}{\tau_{h}}\left(h_{in}\frac{1-{\left(1-E_{0}\right)}^{\frac{1}{h_{in}}}}{E_{0}}-h_{v}^{\frac{1}{\alpha }}\frac{h_{q}}{h_{v}}\right) \end{array}$$

The first two lines in equation (9) are a damped filter (with the resonance frequency of the vasomotor signal, i.e. 0.1 Hz) that converts the neurovascular signal, z, to a vasodilatory signal $h_{s}$. The parameters η and χ in the first equation are the decay rates of the vasodilatory signal and the auto-regulatory feedback term, respectively. Activation of the vasodilatory signal causes alteration in blood inflow $h_{in}$ to the venous compartments, which in turn causes an increase in blood volume $h_{v}$ and a reduction in the level of deoxyhaemoglobin $h_{q}$. The model for blood perfusion dynamics is given by Buxton et al. (1998) Balloon model, in the third and fourth lines in equation (9). The mean rate constant $\tau_{h}$ in the Balloon model is the time taken for blood to pass through the venous compartment (the transit time). The parameter for the blood vessel stiffness is α and is known as Grubb’s coefficient, and $E_{0}$ is the net oxygen extraction fraction at rest, which characterises the fMRI baseline.

#### 2.2.3 fMRI observation model

Finally, the change in blood volume and deoxyhaemoglobin combine to generate the BOLD signal:

(10) $$y_{BOLD}=V_{0}\left\{\;k_{1}\cdot \left(1-h_{q}\right)+k_{2}\cdot \left(1-\frac{h_{q}}{h_{v}}\right)+k_{3}\cdot \left(1-h_{v}\right)\right\}+\varepsilon_{B}$$

with the addition of noise, this is the BOLD signal measured in the scanner. It comprises of physiological and field sensitive parameters, listed in Table 3.

### 2.3 Multimodal estimation procedure

The parts of the model described so far specify a pathway from neuronal activity to MEG and fMRI signals. In this section we set out a novel first level (i.e., within-subject) method for combining these model components and estimating their parameters. The procedure has three stages. First, a typical mass-univariate SPM analysis is performed on the fMRI data, to locate brain regions that evince experimental effects. Second, a DCM for MEG is specified, comprising a neuronal part (Section 2.1.1) and an observation part (Section 2.1.2). The coordinates of the brain regions identified in the fMRI analysis are used as prior constraints on the observation part, which projects neuronal activity to the scalp surface. A DCM is then fitted to the MEG data using the standard variational Laplace scheme (Friston et al., 2003), which provides an estimate of the parameters and the log model evidence (approximated by the negative variational free energy). Next, using the posterior expectations of the neuronal parameters, the DCM is used to generate a posterior predictive neuronal response to each experimental condition; hereafter, *neuronal drive functions*, which form a bridge between the MEG model and the fMRI model.

To clarify this approach, let the simulated electrophysiological response (e.g., pre or post synaptic signals) of population i in region j for the conditions $c_{1},\ldots ,c_{n}$ be denoted by $f_{c_{1}}^{ij}\left(t\right),\ldots .,f_{c_{n}}^{ij}\left(t\right),$ and also assume that the time associated with $l^{th}$ repetition of condition $c_{*}$ in the fMRI experiment is denoted by $t_{l}^{*}$, with total repetitions of the condition $\left|c_{*}\right|$ (i.e., the total number of times that an experimental condition ∗ is shown to a subject is denoted by |∗|). Then the neuronal drives associated with population i in region j to the neurovascular function are calculated as follows:

(11) $$z^{ij}\left(t\right)=\sum_{l=1}^{\left|c_{1}\right|}f_{c_{1}}^{ij}\left(t-t_{l}^{1}\right)+\ldots +\sum_{l=1}^{\left|c_{n}\right|}f_{c_{n}}^{ij}\;\left(t-t_{l}^{n}\right)$$

The $z^{ij}\left(t\right)$ in each region are then combined based on the particular hypothesis about neurovascular coupling. In this paper, the neurovascular drives to the haemodynamic response in region j (each region comprises four populations) were calculated using one of the two general forms:

(12) $$\begin{array}{l} z^{j}\left(t\right)=\text{ ​}\sum_{i=1}^{4}\beta_{ij}z^{ij}\left(t\right)\;\\ z^{j}\left(t\right)=\text{ ​}f_{nc}⊗\left(\sum_{i=1}^{4}\beta_{ij}z^{ij}\left(t\right)\right) \end{array}$$

The first line in equation (12) states that neuronal activity causes the BOLD response instantaneously whereas the second equality introduces a delay and dispersion through the application of a convolution operator that models intracellular calcium dynamics, as in equation (8). We will refer to these two forms as *Direct* and *Delay*, respectively. In equation (12), parameters $\beta_{ij}$s are scalars (for the $i^{th}$ population in region j) that can be constrained to be identical or vary across regions.

Finally, the third step is to use these neurovascular signals as input to the haemodynamic model of responses in each region or source (see the first line of Equation (9)). The parameters and evidence of the haemodynamic models are estimated from the fMRI data using Variational Laplace in the usual way (Friston et al., 2007).

## 3 Illustrative example

### 3.1 Dataset

To illustrate how to apply the methods outlined above, we acquired a dataset from a single subject (right-handed, male, age 30) who performed the same auditory task while undergoing fMRI and MEG on separate days. This experiment was conducted in accordance with the Ethics Committee of University College London, UCL Ethics Ref: 1825/003 (MRI) and Ref: 1825/005 (MEG).

**Fig. 2:**
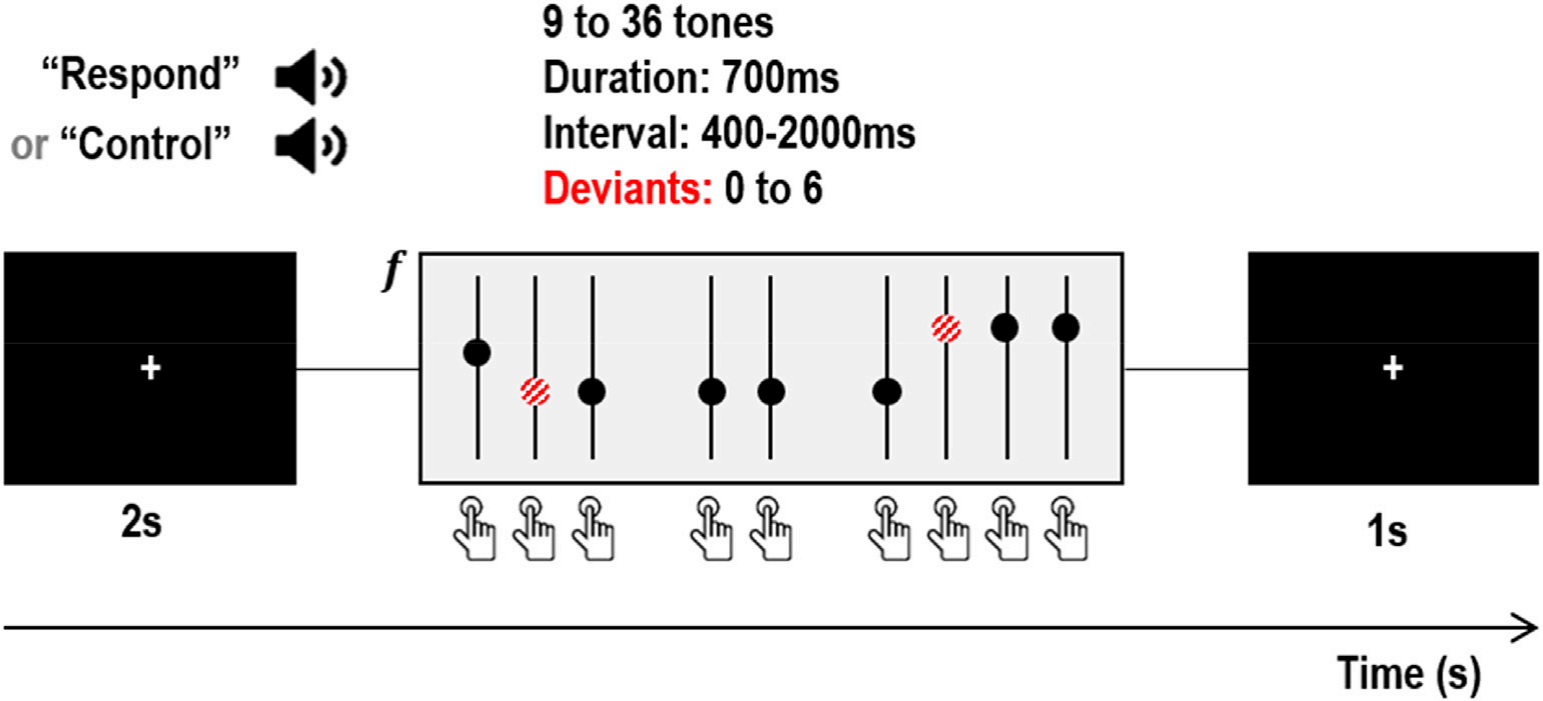
Structure of a single block of the experiment. The subject received an auditory cue, instructing them to respond to auditory tones or control the tones (by pressing a button). After 2s, a series of tones was presented. Deviant tones (red striped circles) differed in frequency from the preceding tone. Whether a tone was a standard or deviant was independent of whether the tone was triggered by the computer or the subject. The block ended with an inter-block interval of 1s. Image credits: Press button by Hea Poh Lin and Speaker by ProSymbols from the Noun Project, CC BY 3.0.

The task was a variant of the auditory roving oddball paradigm (Baldeweg et al., 2004), which has been extensively characterised in patient and control populations using DCM (e.g. Boly et al., 2011, 2012; Dima et al., 2012; Garrido et al., 2008; Rosch et al., 2018). Participants hear a series of ‘standard’ tones of the same pitch (frequency). Occasionally, the tone changes to a new pitch (a ‘deviant’), eliciting neural responses that gradually reduce over the tones that follow, as the deviant becomes the new standard. These neural effects cause marked deviations in the MEG signal (the mismatch negativity, MMN) and we expected there to be concomitant changes in the fMRI signal. We extended the roving oddball task with a second experimental factor of agency. In each block of tones, the auditory stimuli were either produced by the subject (‘control’ condition) or by the computer (‘respond’ condition) as detailed in Fig. 2.

There were therefore two independent experimental factors – surprise (standards vs deviants) and agency (computer-vs human-controlled tones). To maximise fMRI efficiency, the auditory stimuli were arranged into blocks of four types – 1) respond with many deviants 2) respond with few deviants 3) control with many deviants 4) control with few deviants. The computer screen in the MRI scanner and MEG system displayed a white fixation cross on a black computer screen, and the subject was instructed to fixate throughout. We will present analyses focussing on the novel manipulation of agency in a separate manuscript. Here, we used data collected under this task purely to illustrate the estimation of neuronal and neurovascular responses in the auditory hierarchy. The MEG and fMRI datasets were pre-processed using standard procedures in SPM12 (for details, see the supplementary material).

### 3.2 Preliminary fMRI analysis

We used the fMRI data to select regions of interest for the subsequent analyses. Details are provided in the Supplementary Text. In brief, we specified a General Linear Model with regressors (covariates) encoding the onsets of deviants in the control blocks, deviants in the respond blocks, auditory cues instructing the participant of whether they were in a respond or control block, as well as regressors encoding head motion and a constant term. We computed the t-contrast for the main effect of deviants vs standards, thresholded at p ​< ​0.05 family-wise error corrected for multiple comparisons. This identified five regions conventionally included in mismatch negativity studies (Garrido et al., 2008): left and right Heschl’s gyri, left and right planum temporale and right inferior frontal gyrus (IFG). We identified the MNI coordinate of the peak response in each region and extracted a single representative timeseries (the first principal component) from each.

### 3.3 DCM for MEG specification

Pre-processing the MEG data gave rise to four types of event-related potential (ERP), namely standards in respond blocks (SR), deviants in respond blocks (DR), standards in control blocks (SC) and deviants in control blocks (DC). We defined a neuronal (CMC) model comprising a fully connected network (by defining priors on adjacency matrix *A*) to govern dynamics of the four ERP conditions SR, DR, SC and DC in the time interval [0−400]ms post-stimuli. Differences between the four ERPs were characterised by the following between trial effect (BTF) matrix:

(13) $$BTF=\left[\begin{array}{c} SR\;DR\;SC\;DC\\ 0\;\;\;\;0\;\;\;\;\;0\;\;\;\;\;1\\ 0\;\;\;\;0\;\;\;\;\;1\;\;\;\;0\\ 0\;\;\;\;1\;\;\;\;\;0\;\;\;\;0 \end{array}\right].$$

The BTF matrix instructed DCM for MEG to treat the SR condition as the baseline, and to model each of the remaining conditions by adding condition-specific forward and backward *B* matrices (Litvak et al., 2011). The priors for the *B* matrices in this paper were defined such that all extrinsic forward, backward and self-inhibition of neuronal populations were subject to change by the DR, SC and DC conditions. The thalamic inputs, *U*, were received by the lowest level in the cortical hierarchy of our model (left and right Heschl’s Gyrus). The inputs *U* were specified and parameterized by a bell-shaped (Gaussian) function which encoded the delay and dispersion of the neural response to external stimuli. Consistent with other DCM studies of auditory mismatch negativity paradigms (e.g., Garrido et al., 2008, David et al., 2006, and Rosch et al., 2019.), we hypothesised that the effect of stimulation would drive neural activity about 70 ​± ​16 ​ms post stimulus (having said that, one could explore different sets of priors for the input parameters and compare ensuing model evidences associated with them, i.e. using Bayesian model comparison to find the best prior for any specific auditory paradigm). We fitted this model to the MEG data using the eight principal modes of the modelled and observed ERPs as data features (Auksztulewicz and Friston, 2015; Friston et al., 2007). Using the posterior expectations of the neuronal parameters, we then used the canonical microcircuit model to simulate neuronal drives (i.e., posterior predictive expectations) for each of the four experimental conditions.

### 3.4 Neurovascular model specification and comparison

The neuronal inputs to the haemodynamic model were generated from the neuronal drive functions, parameterised according to the hypothesis being tested. Let the simulated neuronal response of population i in region j for the four conditions be denoted by $f_{SR,DR,DC,SC}^{ij}\left(t\right)$. Using equation (11), the neuronal drives associated with population i in region j to the neurovascular function are given as follows:

(14) $$Z^{ij}\left(t\right)=\sum_{l=1}^{\left|DR\right|}f_{DR}^{ij}\;\left(t-t_{l}^{DR}\right)+\sum_{l=1}^{\left|SR\right|}f_{SR}^{ij}\;\left(t-t_{l}^{SR}\right)+\sum_{l=1}^{\left|DC\right|}f_{DC}^{ij}\;\left(t-t_{l}^{DC}\right)+\sum_{l=1}^{\left|SC\right|}f_{SC}^{ij}\;\left(t-t_{l}^{SC}\right)$$

We defined a sample model space that included a set of 16 candidate haemodynamic models covering a number of biologically informed hypotheses about the nature of neurovascular coupling. These models varied according to four model attributes or factors:

Q1: How should neurovascular coupling be parameterised? We considered three options, regarding whether the haemodynamic part of the model should be driven by:
  • collaterals from presynaptic inputs to each population, with separate parameters for each population
  • collaterals from presynaptic inputs to each population, grouped into excitatory, inhibitory and extrinsic collaterals (Friston et al., 2017)
  • postsynaptic neuronal drive (f functions in equation (11))
Q2: Should distal neuronal sources exert changes on the regional BOLD response? In other words, should haemodynamics be driven by local neuronal populations only, or additionally by exogenous inputs from other regions?
Q3: Should neurovascular coupling parameters be region-specific or equal for all regions (β in equation (12))?
Q4: Should a Direct or Delay model governing the dynamics of astrocyte responses be used (selection of the first or second equality in equation (12))? This addresses the delays associated with the release of vasoactive agents (e.g., intracellular calcium).

These four questions underwrite 16 candidate models, listed in Table 5. We then estimated the parameters and evidence (free energy) for each of the models using a standard variational Laplace scheme (Friston et al., 2007). To address each experimental question, we grouped the candidate models into families and compared them using family-wise Bayesian model comparison (Penny et al., 2010). Finally, we used Bayesian model comparison over the entire model space to find the most parsimonious explanation for the origin of the BOLD response in our dataset.

**Table 5:** Model space design to investigate function of neurovascular coupling.

Model	F1: Parameterisation	F2: Distal inputs?	F3: Region-specific?	F4: Direct vs Delay
1	Pre	Yes	Yes	Direct
2	Pre	No	Yes	Direct
3	Pre	Yes	No	Direct
4	Pre	No	No	Direct
5	Post	N/A	Yes	Direct
6	Post	N/A	No	Direct
7	Pre (Friston et al., 2017)	Yes	No	Direct
8	Pre (Friston et al., 2017)	No	No	Direct
9	Pre	Yes	Yes	Delay
10	Pre	No	Yes	Delay
11	Pre	Yes	No	Delay
12	Pre	No	No	Delay
13	Post	N/A	Yes	Delay
14	Post	N/A	No	Delay
15	Pre (Friston et al., 2017)	Yes	No	Delay
16	Pre (Friston et al., 2017)	No	No	Delay

∗ Factors F1–F4 correspond to the factors of the experimental design described in Section 3.4.

### 3.5 Results

**Fig. 3:**
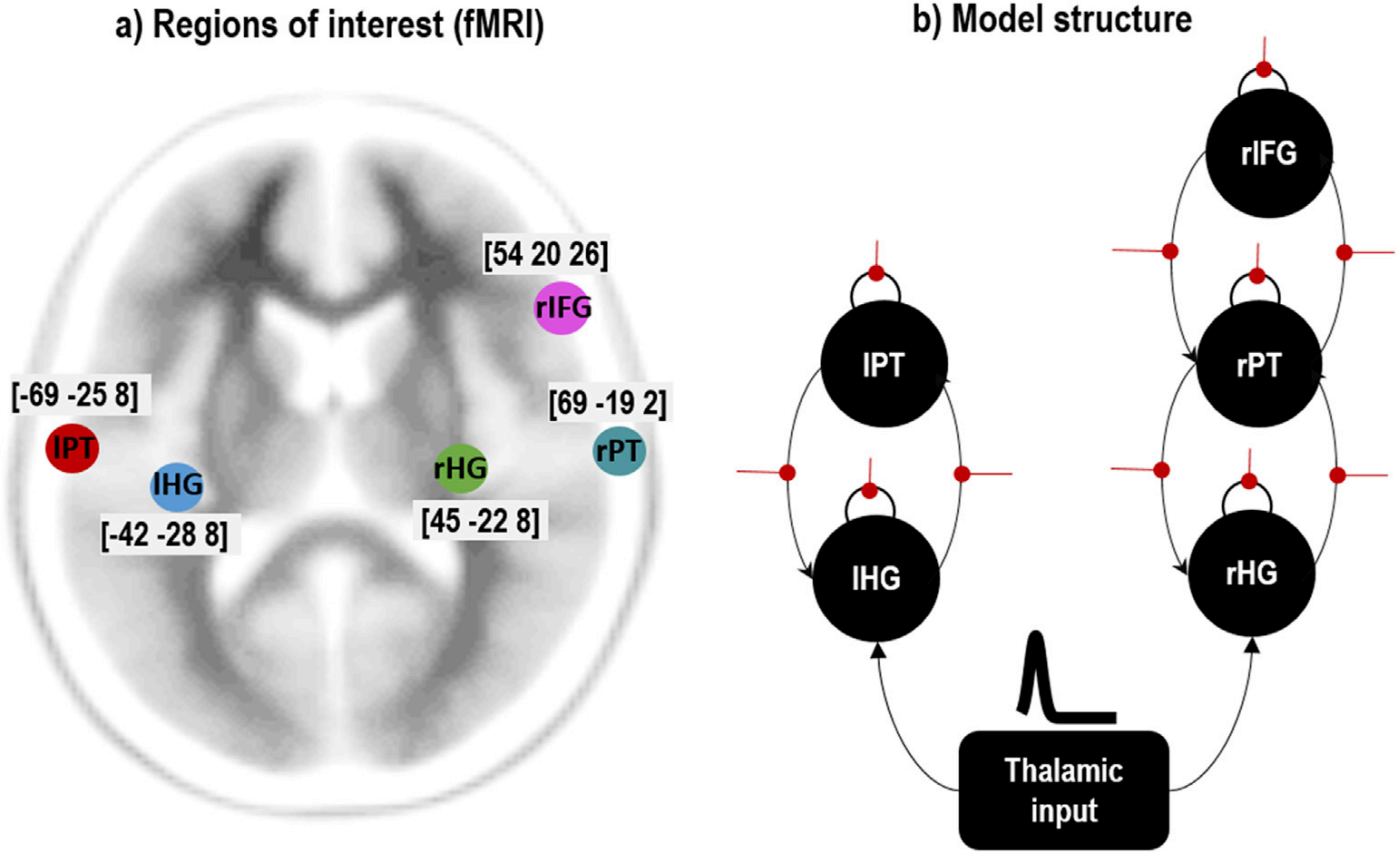
Region of interest selection and DCM network structure. a) Five neuronal sources activated during the fMRI experiment, as identified using a mass univariate analysis. These were left and right Heschl’s gyrus (lHG, rHG), left and right planum temporale (lPT,rPT) and right inferior frontal gyrus (rIFG). Peak MNI coordinates, used as priors for MEG source localisation, are shown. b) Structure of the neuronal part of the DCM. Each large black circle is a canonical microcircuit (CMC), extrinsic connections between regions are shown as curved black lines, and connections that were subject to change – from one condition to another – are indicated with straight red lines.

We first used the fMRI data to locate brain regions responding to the main effect of deviants versus standards. As hypothesised, this included five regions typically found in the oddball paradigm, shown in Fig. 3a.

**Fig. 4:**
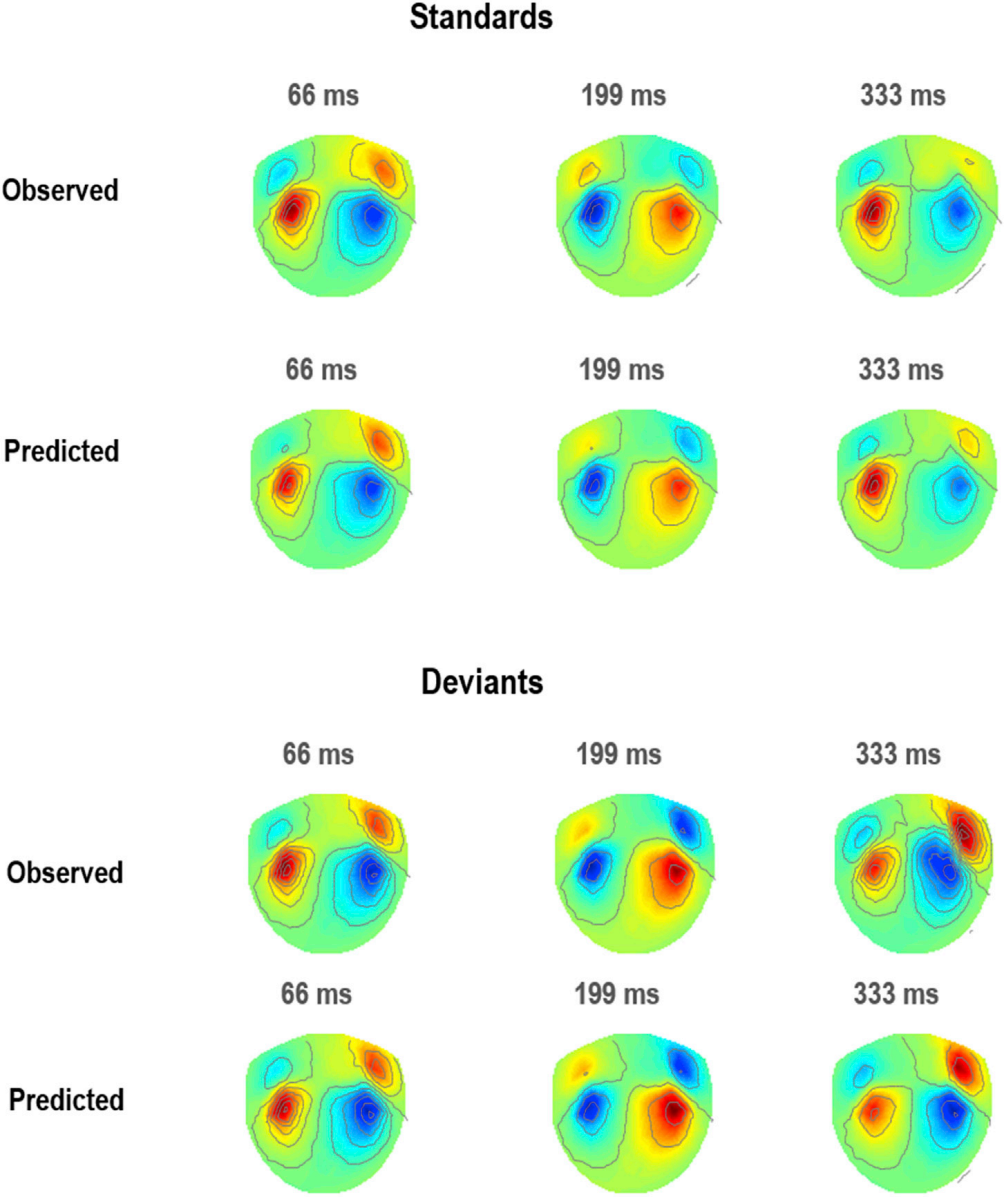
DCM for MEG results. This figure shows scalp map projections of observed and predicted responses for two conditions; namely, standard and deviant tones (in the respond blocks only).

Next, we used the coordinates of these five regions as priors for source localisation in DCM for MEG. We specified a DCM, as shown in Fig. 3b, where each brain region or source (large black circle) was a canonical microcircuit. We fitted this model to the MEG data. Fig. 4 shows the scalp maps associated with the prediction of the model and the observed data over the time course of a trial. A close correspondence between the predicted and real data is apparent.

**Fig. 5:**
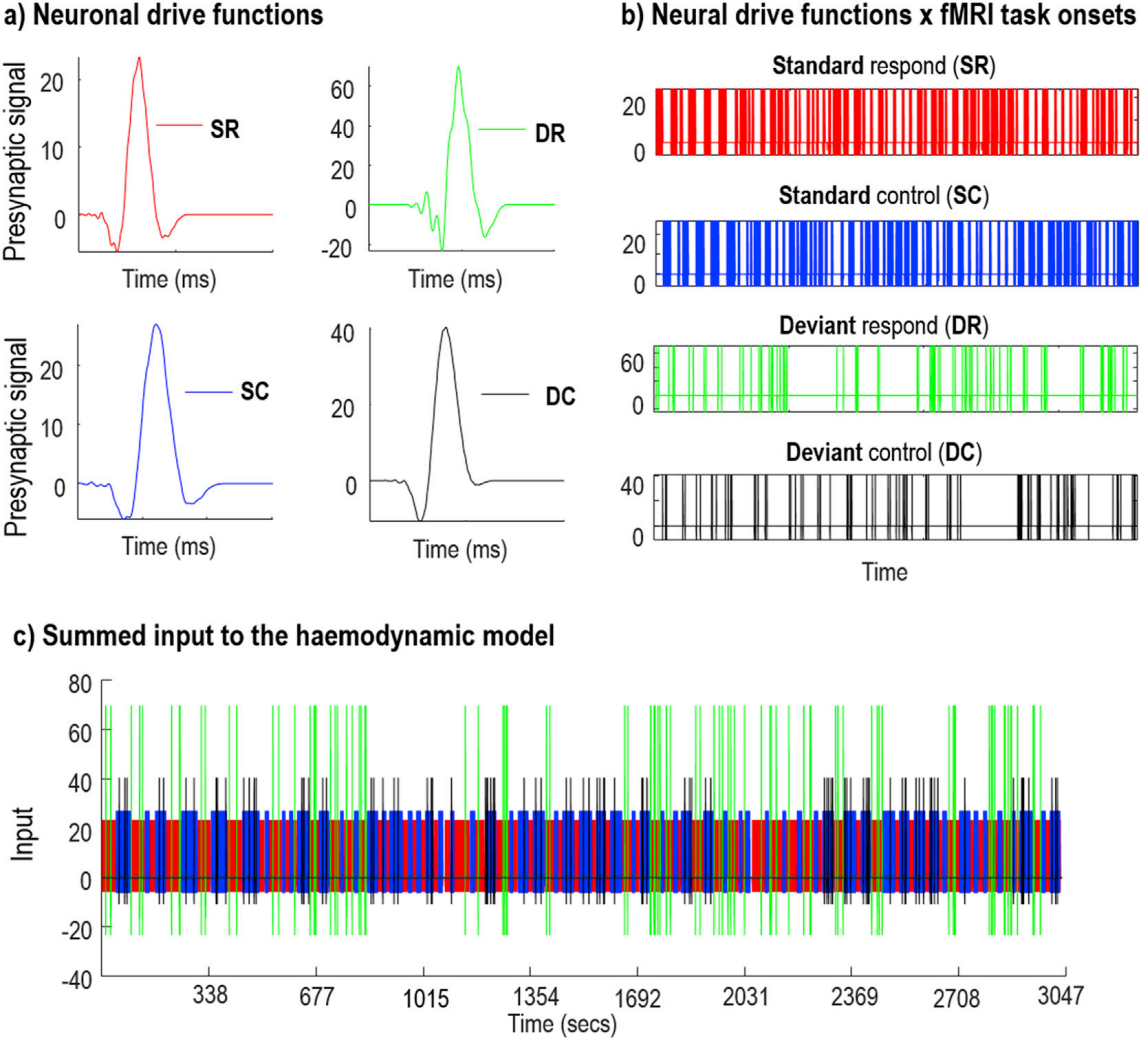
Simulated neuronal drive associated with one neuronal population. DCM for MEG was first used to infer the neuronal parameters of CMC models. a) The ensuing neuronal parameters were used to generate condition specific neuronal responses (e.g., pre-synaptic signals). b) To generate the input for the haemodynamic model, the neuronal drive functions were convolved (or shifted in time) with the onset of each trial of the fMRI experiment. c) All condition specific neural responses were then summed to generate the neuronal drives to neurovascular coupling units. This was repeated for each neuronal population and brain region.

**Fig. 6:**
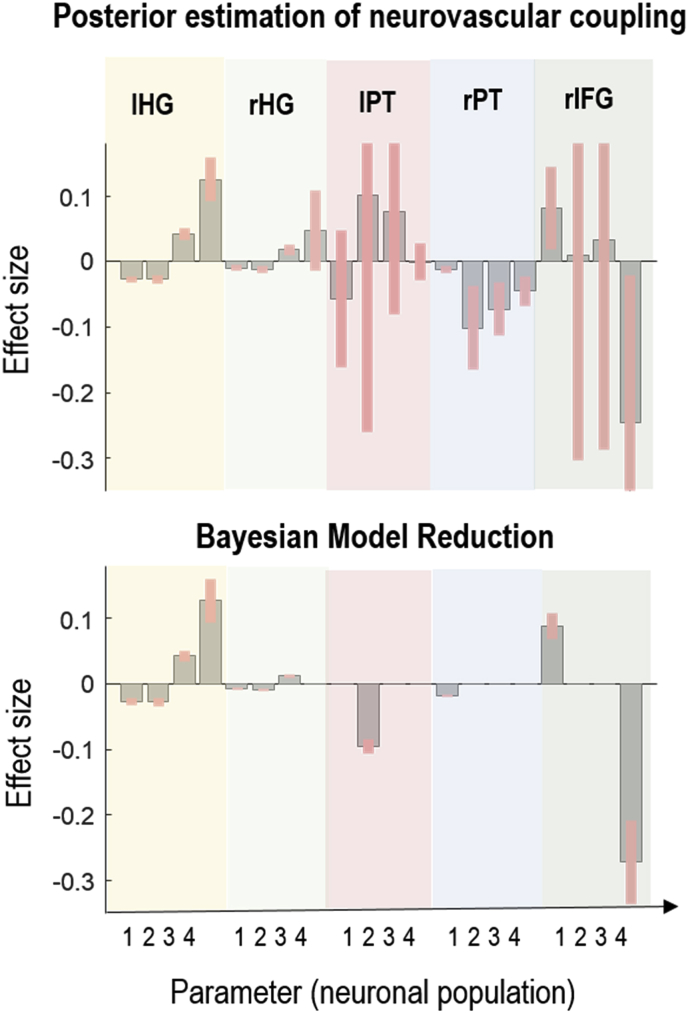
Estimated neurovascular parameters. The plots show posterior estimates of the neurovascular coupling parameters β that best accounted for the multimodal data and BMR analysis of estimated parameters that elucidate key parameters governing dynamics of data. The grey bars are the expected values and the pink error bars are 90% credible intervals. Each group of 4 ​bars corresponds to parameters quantifying the contribution to the neurovascular coupling by: spiny stellate (SS), superior pyramidal (SP), inhibitory interneurons (II) and deep pyramidal (DP) cells. The titles indicate the brain regions: left Heschl’s gyrus (lHG), right Heschl’s gyrus (rHG), left planum temporale (lPT), right planum temporale (rPT), right inferior frontal gyrus (rIFG). 1

**Fig. 7:**
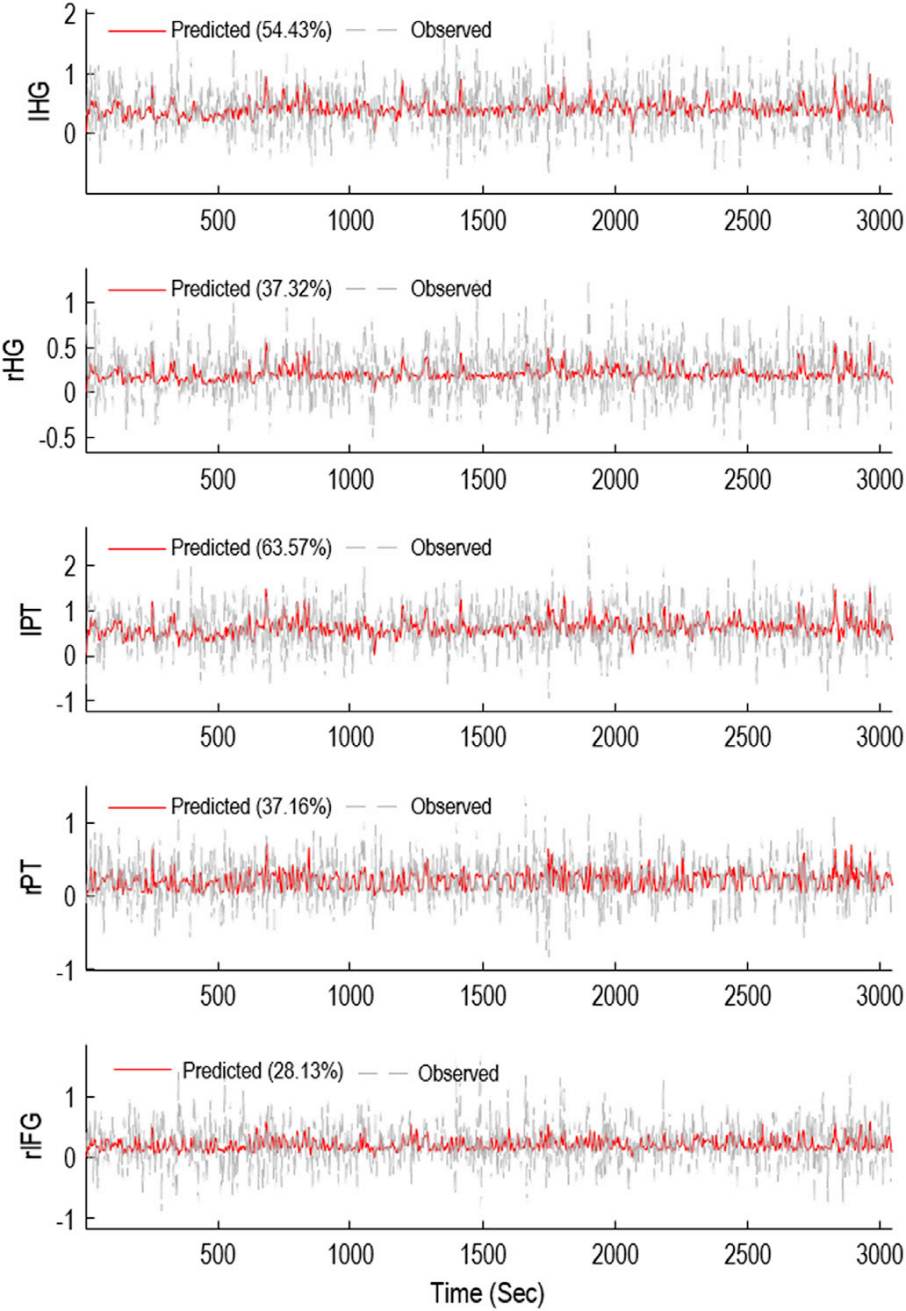
Model prediction and observed data. Predicted BOLD time series associated with the instantaneous region-specific model of neurovascular coupling mechanisms, driven by regional presynaptic signals, as well as observed fMRI time series are shown. The vertical axis labels are associated with the five brain regions: left Heschl’s gyrus (lHG), right Heschl’s gyrus (rHG), left planum temporale (lPT), right planum temporale (rPT), right inferior frontal gyrus (rIFG). Numbers in the legend denote explained variance (%).

We then used the posterior neuronal estimates to simulate pre/postsynaptic potentials associated with the four experimental conditions – i.e. to generate neuronal drive functions. These are shown in Fig. 5a for the inhibitory population in the IFG region (the rest of the neuronal drives were calculated in a similar way). These condition-specific responses were then aligned with the associated condition-specific stimulus onsets in the fMRI experimental design (equation (11) and Fig. 5b). Neuronal drives associated with each source were then summed (and in some models filtered to replicate delay dynamics of neurovascular coupling) to generate the neurovascular drive to the haemodynamic model (equation (14) and Fig. 5c).

As detailed in Section 3.4, we specified and estimated 16 candidate haemodynamic models, which varied in their mechanisms of neurovascular coupling according to four model factors. We then divided the models into ‘families’ according to each factor and performed a series of family comparisons. For this exemplar single subject analysis, the results of Bayesian model comparison showed that neurovascular coupling was best explained (with a posterior confidence approaching 100% for each comparison) as:

(i) driven by collaterals from presynaptic input, separately parameterised for each neuronal population, rather than presynaptic input grouped into excitatory/inhibitory/exogenous connections or postsynaptic input
(ii) driven by local neuronal projections *without* afferent input from distal regions
(iii) separately parametrised on a region-specific basis, rather than having shared weights for each condition and neuronal populations across brain regions
(iv) having a direct form of model governing the dynamics of astrocyte responses, as opposed to a delayed effect.

The overall winning model in our sample model space, with a log Bayes factor of 7.67 compared to the next best model, suggested that this subject’s BOLD response could best be explained by instantaneous local presynaptic neuronal activity, with region-specific parameterisation of neurovascular coupling. Fig. 6 shows the estimated neurovascular coupling parameters from this model, with parameters not contributing to the model evidence pruned using Bayesian model reduction. For each parameter, Bayesian model reduction was also used to test the hypothesis that the parameter was present vs absent (i.e. non-zero vs zero). In this plot, each group of 4 ​bars are the estimated contribution of each neuronal population (SS, SP, II, DP) to the haemodynamic model. In all five regions there were parameters which deviated confidently from their prior expectation of zero, confirming that the synaptic activity estimated from the MEG data captured variance in the fMRI data (explained variance per region: 53%, 37%, 64%, 37% and 28%). Fig. 7 shows the prediction of the winning model and the observed fMRI time series of the five regions.

Readers should note that this example is only intended to illustrate how to apply the proposed method, therefore the results should not be generalised from the exemplar subject, with this specific experimental paradigm. To make inferences about typical and atypical neurovascular coupling, group studies would be necessary, with the appropriate between subject modelling and model comparison.

## 4 Discussion

### 4.1 Methodology

The novel contribution of this work is to establish a relatively straightforward multi-modal DCM procedure that flexibly connects laminar-specific neural mass models, which are fitted to electrophysiological data, with neurovascular models, which are fitted to fMRI data, via simulated neuronal drive functions. Together, these form a complete generative model of the BOLD signal, which enables hypotheses about neurovascular coupling to be tested efficiently using Bayesian model comparison and reduction. The neuronal drive functions act as a bridge between the fMRI and MEG modalities, enabling multi-modal analyses to be conducted with any of the neural mass models implemented within the DCM framework. We addressed the difficult parameter identification problem inherent in having a single generative model of both BOLD and electrophysiological recordings by separately estimating neuronal parameters using MEG data, and neurovascular/haemodynamic parameters using fMRI data. This can be seen as a simple form of Bayesian belief updating, in which the posterior estimates based upon MEG data are used as precise priors for models of haemodynamic responses, which share a common set of neuronal parameters. Crucially, we can leverage this form of Bayesian belief updating using ‘off the shelf’ dynamic causal models for both modalities. The only things we need to add are neuronal drive functions that link the modality-specific DCMs. The proposed approach may offer new insights into the source of the BOLD response in the healthy and pathological brain and is available through the SPM software.

As noted in the introduction, the purpose of this paper is to introduce an analytic procedure – not to draw any definitive conclusions about the nature of neurovascular coupling or how haemodynamics are affected by demographic or diagnostic factors. We therefore elected to present a single case study. This analysis can be generalised to group studies using hierarchical or parametric empirical Bayes for dynamic causal modelling (Friston et al., 2015, 2016). In principle, this provides an opportunity to infer the nature of haemodynamic coupling that is conserved over subjects. However, this particular application was not our focus, largely because a detailed mechanistic understanding of haemodynamics would be better informed by more invasive data (probably from animal studies). Rather, our goal was to provide efficient estimates of haemodynamic parameters from non-invasive (human) data, enabling researchers to investigate factors like age and pathology (e.g., migraine, Alzheimer’s disease) on haemodynamic parameters – in a way that is not confounded by uncertainty about changes in neuronal coupling and intrinsic circuitry. In this light, the current paper can be regarded as a foundational (technical) description of the methodology that could pave the way to addressing questions about between-subject effects on haemodynamic parameters (under a particular model of neurovascular coupling). This kind of application speaks to the underlying motivation for combining electromagnetic and haemodynamic data. In short, the principal advantage of multimodal fusion in this paper is the opportunity to estimate and quantify haemodynamics per se. The extra information afforded by MRI data about neuronal parameters is limited, given an appropriate model of electromagnetic responses. The key thing that the MRI data brings to the table is the ability to quantify neurovascular coupling given (MEG or EEG based) estimates of neuronal coupling, on a per subject basis. In this setting, the procedures outlined above are aimed explicitly at disambiguating changes in neuronal and haemodynamic coupling, when quantifying age and disease-related neurophysiology.

One might ask what the advantages are of acquiring fMRI and M/EEG data in separate sessions – as opposed to simultaneously. Clearly, simultaneous acquisition has the benefit of modelling the electromagnetic and haemodynamic responses to the same neuronal generators. However, from a statistical perspective there are advantages to separate acquisition protocols. These rest upon the fact that the efficiency of the design can be optimised for each modality separately. This is particularly prescient given that the haemodynamic response function imposes particular constraints on experimental design for fMRI, which would be inappropriate for an EEG paradigm. For example, one can use many more EEG trials under a separate acquisition protocol. Assuming a stereotypical neuronal response for each trial type therefore enables an efficient estimation of neuronal (and haemodynamic) model parameters, via the use of trial averages.

#### 4.1.1 Relation to other methods

The approach set out here can complement well-established empirical methods for measuring cerebrovascular reactivity; namely, CO2 challenges (Maggio et al., 2014; Salient et al., 2014). These procedures enable blood flow to be modulated and quantified in vivo; however, they do not enable one to estimate the underlying neuronal responses to various stimuli. Furthermore, these methods may not be appropriate in all situations. For example, where the study of certain clinical populations precludes the use of gas challenges. Therefore, a non-invasive method that relies only on BOLD contrast, such as that described here, could be more practical. Additionally, using electromagnetic responses that are generated directly from neuronal (depolarisation) sources allows one to compare neurovascular models that map from neuronal responses to haemodynamic and metabolic responses in a more efficient manner.

The multimodal dynamic casual modelling approach presented in this paper (for investigating neurovascular mechanisms) can be compared against other modelling and simulation techniques. Pang et al. (2017) considered different models of neurovascular coupling, each of which drove a canonical haemodynamic response function (HRF). They fitted these models (where each formulated a different neurovascular mechanism with a common HRF) to BOLD time series and used goodness of fit criteria for model selection. Schirner et al. (2018) used structural imaging for inferring effective connectivity and simulated data from a hemodynamic model that was driven by EEG source activity (under the hypothesis that excitatory activity, as reflected by EEG, perfused blood flow) to replicate BOLD responses similar to real fMRI data.

From a technical standpoint, using variational Bayesian techniques (inferring parameters by optimising free energy) is superior to maximum likelihood or goodness of fit (Bishop, 2006). This follows because fitting models based only on their accuracy fails to account for model complexity and precludes generalisability. In addition, model estimation using dynamic casual modelling allows for estimation of the posterior probability of parameters and model evidence, which is necessary for model selection based on Bayesian model comparison (Jafarian et al., 2019). This allows for testing and comparison of several hypothesis about different mechanisms of neurovascular coupling. The use of multimodal data provides complementary constraints on parameter estimation that afford a greater efficiency – in terms of parameter estimation and model comparison – then using a single (i.e., fMRI) modality (Wei et al., 2020). In short, our proposed multimodal approach could complement existing fMRI DCM studies elucidating, for instance, neural and haemodynamic contributions to aging (Tsvetanov et al., 2016).

### 4.2 Potential applications

To illustrate the type of hypotheses that can be addressed using this approach, we used Bayesian model comparison to address four experimental questions in a single subject MEG/fMRI dataset. Our model space could be applied directly to data from a group of subjects, or it could easily be modified in order to accommodate different hypotheses about neurovascular coupling (please see the software note for more information). In practice, we expect that a model space such as this would be used to identify a parsimonious model that was apt for a group subjects, before being used to quantify subject specific differences in model parameters, for example due to aging or disease.

To illustrate this kind of model comparison, we asked whether presynaptic or postsynaptic neuronal activity mediated haemodynamic responses. This sort of question speaks to the findings of Attwell and Iadecola (2002) and Logothetis (2003, 2008), who concluded that mean neuronal firing rates (presynaptic signals) are largely responsible for the BOLD response in humans.

The second question was whether extrinsic collateral afferents from distal regions contribute to haemodynamics, or whether neurovascular coupling should be considered a purely local phenomenon. Bayesian model comparison suggested that local neuronal activity provided the best explanation for the BOLD response, as is assumed, for example, in mass-univariate (SPM) analysis or Dynamic Causal Modelling (DCM) for fMRI.

The third question in this illustrative model space was whether the contribution of neuronal populations to the neurovascular units was identical across brain regions or region-specific. Model comparisons of this sort could establish whether neuronal contributions to neurovascular mechanisms are region-specific (Devonshire et al., 2012), or indeed distinct across cortical layers (Goense et al., 2012 & 2016).

The fourth question we asked was whether the BOLD signal was best explained as being driven by a Direct (scaling only) or Delay (scaling and delay) model of neurovascular coupling. This question was motivated by studies in animal models, suggesting a delay between neuronal activity and the BOLD response due to elevation of intracellular calcium in astrocyte collaterals (Rosenegger and Gordon, 2015). We used a lumped linear second-order model, which can be inferred efficiently using fMRI data. The ensuing model comparison addressed questions about whether instantaneous electrophysiological fluctuations induce BOLD responses directly, as reported in Logothetis (2003).

The proposed framework may be particularly useful for studying processes that effect both neuronal and haemodynamic responses. For instance, it could be used to model effects of aging (D’Esposito et al., 2003) in cognitive paradigms, where aging would be expected to not only affect neuronal responses, but also the stiffness of blood vessels, quantified by Grubb’s exponent in the haemodynamic model (see Equation (8)) and/or delays in the model neurovascular coupling. To facilitate this, multimodal DCM could be combined with the parametric empirical Bayes (PEB) (Friston et al., 2016), to test for differences in neurovascular and haemodynamic parameters between young and old age groups. The approach in this paper may also be useful for characterising experimental manipulations for which neurovascular function alone is altered. For instance, the action of a particular intervention such as diazoxide is predominantly on neurovascular coupling, with little effect on neuronal dynamics (Pasley, 2008). In summary, the current modelling initiatives, together with PEB for random (between subject) effects analysis, are well placed to characterise and elucidate neurovascular physiology.

Finally, an interesting application could address the genesis of the negative BOLD signal. To do so, one would start by designing a paradigm (e.g. Klingner et al., 2011; Huber et al., 2014) to elicit both positive and negative BOLD responses. Using multimodal DCM and Bayesian model comparison, one could test hypotheses about neurovascular mechanisms that might induce negative BOLD (Valdes-Hernandez et al., 2018). Interesting questions might include (i) do BOLD responses result from positive/negative neuronal drive signals as introduced in this paper? (ii) Does neuronal inhibition significantly contribute to the negative BOLD (Shmuel et al., 2006; Bernal-Casas et al., 2017)?

### 4.3 Limitations and further development

A common issue in non-simultaneous multimodal paradigms is the possibility of different underlying generators of neuronal responses for each modality (Wibral et al., 2010). For the example analysis in this paper, the use of MEG data to inform the characterisation of fMRI data rests explicitly on having a common neural model that can generate both modalities, which shares the same neuronal parameters and architecture (see Hall et al., 2014a, Hall et al., 2014b). In other words, we assume that the neuronal responses in the two recordings – under the same paradigm – are generated in the same way. However, if quantifying neuronal plasticity over time were of interest, one could collect MEG and fMRI data for several days and perform multi-modal DCM at each time point. Then, the parameters of the MEG-informed haemodynamic model (associated with each day) could be entered into a second level analysis to test for commonalities and differences over time. For example, one could test for differences between scanning in the morning and evening, or for a parametric effect of the number of days between recordings. Given that we have illustrated the procedure using a single dataset, we could not illustrate tests for session to session variability. However, between session (or subject) variability in model parameters is generally assessed using hierarchical models, known technically as Parametric Empirical Bayes (Litvak et al., 2015). This is an established technology for between session and between subject effects in the parameters of dynamic causal models and – in principle – would be straightforward to apply in the current setting. In other words, having established the model of neurovascular coupling with the greatest evidence at the between session (or subject) level, one can then quantify the between session (or subject) variability in model parameters.

The approach described here affords the opportunity to investigate how (weighted) laminar-specific neuronal activity contributes to a single measurement (per region) BOLD signal. Therefore, a key limitation of the model is the assumption of a single haemodynamic compartment. This could be improved by using laminar fMRI data. In fact, neural vasculature has a well-studied spatial arrangement in the cortical depth, which was modelled in the DCM framework by Heinzle et al. (2016). This modelling approach could be incorporated in the approach described here, to better account for differences across laminae due to vasculature. Furthermore, as high spatial resolution fMRI data becomes more readily available – with the rollout of 7 ​T scanning – the question arises of how to make use of these data to inform estimates of neurovascular coupling parameters. There is considerable interest in associating the BOLD response with specific layers of the cortical column, and the laminar specificity of forward and backward connections (e.g. Scheeringa and Fries, 2017; Lawrence et al., 2017; Duyn, 2012; Scheeringa et al., 2016). Typically, laminar fMRI involves dividing the cortical depth into several layers and extracting time series from each layer. Incorporating these laminar specific measurements into the framework presented here could, in principle, be achieved by incorporating a mapping between neuronal activities corresponding to cortical layers and the laminar haemodynamic model.

It is worth reiterating that the current approach is flexible in the sense that one can select different models (or different priors) that best accommodate the scientific question at hand (see the review by Huneau et al., 2015 for different examples). The selection of neuronal and haemodynamic models (and their priors) – for the exemplar analysis in this paper – was motivated by the fact they are well established in the literature, and are readily available within the SPM software. Nevertheless, as physiological understanding and imaging fidelity improve, there are new opportunities for development of the models themselves. In the example presented here, we used the classic model of Buxton et al. (1998) to generate predicted haemodynamic responses. However, alternative haemodynamic models could be implemented and compared based on their evidence, to address particular questions of interest. For instance, there is significant interest in elucidating the mechanisms that give rise to the BOLD post-stimulus undershoot (PSU) (Van Zijl et al., 2012) as well as differences in the PSU between experimental conditions and people. Characterising this phenomenon calls for models that distinguish the neural, neurovascular and haemodynamic contributions to the observed fMRI signal – the statistical efficiency of which can be improved by the use multi-modal data, such fMRI and EEG/MEG (Wei et al., 2020). A recent example of a promising dynamic haemodynamic model, which could be implemented in the DCM framework, explains differences in the PSU (and other transient data features) between cortical laminae, by explicitly encoding haemodynamic flow through ascending veins (Havlicek and Uludağ, 2020). The evidence for a model such as this could be compared against the model used here and the optimal one selected for a particular application. Note, however, that if the experimental interest is primarily about condition-specific neural/neurovascular effects, the choice of haemodynamic model may have a limited influence on the results (e.g. as found by Havlicek et al., 2015). This is because there is, to a large degree, conditional independence between neural and haemodynamic parameters (Stephan et al., 2007), a situation further improved by the use of multi-modal data (Wei et al., 2020). This suggests that different contributions to the data could be quantified efficiently. Another example would be for modelling metabolic activity; i.e. the usage of glucose induced by excitatory and inhibitory activity. For this, one may consider using inhibitory and excitatory neuronal drive functions, introduced here, as the inputs to the model by Sotero and Trujillo-Barreto (2007, 2009).

Finally, the priors for the model parameters (for both CMC and haemodynamic models) can also be updated based on empirical studies. As an example, SPM assumes a prior for the resting blood volume $V_{0}$ of 8%. Since the introduction of this model, studies have generally found a lower value (e.g. 5%) (Leenders et al., 1990). Changes in prior assumptions can be implemented easily by changing the appropriate Matlab function encoding the observation model (e.g., spm_gx_hdm.m). Priors for the parameters of CMC model can also be updated. For example, the particular parameter setting of the sigmoid function in the CMC model (e.g., one may consider updating firing thresholds to 6 mV or treating it as free parameter) to better accommodate biological plausibility (the associated function is spm_dcm_cmc_tfm.m). In summary, we hope the statistical tools presented here will prove useful, for both the ongoing development of neurovascular models and the application of these models for testing hypotheses using multi-modal data.

## 5 Software note

Tools for conducting the analysis procedure presented in this paper are included in SPM12 software. The key function for multimodal DCM inversion is spm_dcm_nvc.m. Input to this function is a cell array that includes: (i) SPM analysis of fMRI data, (ii) extracted fMRI time series, (iii) DCM for M/EEG, (iv) options for how neuronal responses excite neurovascular coupling and how neuronal vascular coupling should be modelled, and (v) a matrix that defines whether any neuronal activity should be excluded from the study (e.g. excluding pre/post synaptic inhibitory activity from the neuronal drive functions). The model options defining the interface between neuronal and neurovascular coupling should be defined as a Matlab cell array. The first entry of the cell indicates that the BOLD signal can be induced by pre- (‘pre’), post- (‘post’) synaptic signals or decomposed presynaptic signals (‘de’) (Friston et al., 2017). The second entry defines whether for different brain regions, the neurovascular coupling model has the same (‘s’) or different (‘d’) parameters. The third entry is to select whether extrinsic neuronal activity (‘exc’) or intrinsic neuronal activity (‘int’) induces regional BOLD signals (when using the option ‘post’, this option should be set to ‘na’). For instance, the model option M ​= ​{‘pre’, ‘s’, ‘int’} states that the presynaptic neuronal drive (excluding extrinsic neuronal drives) induces a model of neurovascular coupling that has the same parameters across all regions.

To exclude any neuronal drive from the fMRI study, 4 ​× ​1 vectors with an entry of one (present) or zero (exclude) can be defined (the first entry is associated with superficial pyramidal cells, the second entry with inhibitory interneurons, the third entry with excitatory interneurons and the fourth entry is associated with deep pyramidal cells). For instance, if we wanted to exclude an inhibition signal from the fMRI inversion, we could define vector O ​= ​[1 0 1 1]. The option for excluding some of the neuronal drives allows one to specify models that emulate signalling mechanisms such as glutamate release (typically from excitatory populations) that may dilate capillaries directly by relaxing pericytes (Hall et al., 2014a, Hall et al., 2014b). In effect, one can evaluate the evidence for models in which excitatory signals to neurovascular coupling (potentially mediated by glutamate) have distinct effects on the BOLD response compared to inhibitory populations.

Functions that are called by the estimation function (spm_dcm_nvc.m) include: (i) spm_dcm_nvc_fmri_priors.m, which can be used to define priors for neurovascular parameters as well as the haemodynamic response function; (ii) spm_dcm_nvc_specify.m, which takes the SPM. mat file for fMRI analysis and creates experimental input time series for fMRI inversion (this routine also defines necessary parameters for DCM inversion); (iii) spm_dcm_nvc_nd.m, which uses estimated neuronal parameters from DCM for ERP and generates a neuronal drive function over different experimental fMRI inputs; and (iv) spm_nvc_gen.m which generates a BOLD signal prediction from the scaled summed of neuronal drives. Inputs to spm_dcm_nvc_nd.m are a DCM for M/EEG and experimental inputs for fMRI. This function uses (i) spm_fx_cmc_tfm_gen.m to create (decomposed) presynaptic signals (with or without external neuronal drive), (ii) spm_dcm_nvc_rep.m, which replicates the neuronal signals over fMRI trials and (iii) spm_gen_par.m, which generates condition-specific neuronal parameters from DCM for M/EEG.

## CRediT authorship contribution statement

**Amirhossein Jafarian:** Conceptualization, Methodology, Software, Validation, Formal analysis, Writing - original draft, Data curation, Visualization. **Vladimir Litvak:** Writing - review & editing, Supervision. **Hayriye Cagnan:** Writing - review & editing, Supervision, Investigation, Conceptualization. **Karl J. Friston:** Conceptualization, Methodology, Writing - review & editing, Supervision, Funding acquisition. **Peter Zeidman:** Conceptualization, Methodology, Writing - review & editing, Supervision, Project administration, Investigation.

## Footnotes

1 The results in Fig. 6 were reported in a provisional form – as part of a tutorial/review in Jafarian et al., (2019).
