# Supplementary material for: Comparing dynamic causal models of neurovascular coupling with fMRI and EEG/MEG
Source: Neuroimage. 2020 Aug 1;216:116734. doi: 10.1016/j.neuroimage.2020.116734 (PMC7322559; doi:10.1016/j.neuroimage.2020.116734)
Supplement: Multimedia component 1 [file mmc1.docx]

**Supplementary material**

*Comparing dynamic causal models of neurovascular coupling with fMRI and EEG/MEG,*

*Jafarian et al.*

## Supplementary methods

### Participants

We scanned a single subject (male, right-handed, age 30) performing the same task under fMRI and MEG. This experiment was conducted in accordance with the Ethics Committee of University College London, UCL Ethics Ref: 1825/003 (MRI) and Ref: 1825/005 (MEG).

### Task

This study used a novel version of the auditory roving (mismatch negativity) oddball paradigm (Garrido at al. 2008) which included an additional factor of agency, such that the auditory stimuli were produced by the subject (self) or by the computer (other). The subject alternated between responding with button presses to a series of computer-generated tones, and generating a series of tones himself using button presses. During the alternation of subject- and computer-generated sequences, the tone of the stimulus changed sporadically, producing oddball responses – that resolved over trials as the new tone became the standard.

There were two experimental factors – surprise (standards vs deviants) and agency (computer- vs human-controlled tones). To maximise fMRI efficiency, the subject was presented with auditory stimuli arranged into blocks, and there were four block types – 1) respond with many deviants 2) respond with few deviants 3) control with many deviants 4) control with few deviants. The experiment was entirely auditory – the computer screen in the MRI scanner displayed a white fixation cross on a black computer screen, and the subject was instructed to fixate throughout.

The structure of a block is summarised in Figure 2 of the main text of the paper. At the start of computer-controlled blocks, the subject heard the auditory cue ‘respond’. A sequence of 70ms auditory tones was then presented with irregular intervals between them, ranging from 400ms to 2000ms. The subject pressed their button each time they heard a tone. The human-controlled blocks started with the auditory cue ‘control’. The subject controlled the onset of the tones, and were instructed to press their button to trigger tones at times of their choosing. They were trained to keep the pace of button-pressing similar to the computer-controlled blocks, but to freely alter the time between individual tones as desired. To ensure that there were no systematic differences in the intervals across conditions, the intervals between tones in the computer-controlled block $n$ were taken from human-controlled block $n-2$ and their order was reversed, to reduce the possibility of the timing sequence being recognisable.

The tones within a block had auditory frequencies 500Hz, 600Hz, 700Hz and / or 800Hz. A ‘deviant’ tone was one which differed in frequency from the previous tone, whereas a ‘standard’ tone had the same frequency as the previous tone. The first tone in a block always had the same frequency as the last tone of the previous block. For the ‘many deviant’ blocks, the number of deviants was sampled from a Poisson probability density function $f$ and calculated using the following equation:

$$n=1+f(x|\lambda=1.5)$$

Where $x$ was a random vector. In the ‘few deviant’ blocks, all but two blocks per run had zero deviants (i.e. all standards), and two blocks had one deviant.

The number of tones in each block was varied, to reduce anticipation of the end of the block. In the many deviant blocks there were 28, 30, 32, 34 or 36 tones, and in the few deviant blocks there were 9, 10, 11, 12 or 13 tones. By reducing the number of tones in the few deviant blocks, the design efficiency was improved (by avoiding unnecessary over-sampling of the standard tones).

The subject was instructed on how to perform the task (see Supplementary text: subject Briefing) and performed a practice run in front of a desktop PC, consisting of 4 blocks. They were then positioned in the MRI scanner and performed a further practice run of 4 blocks. They then performed 3 runs of the task while undergoing fMRI, where each run consisted of 860 tones divided across 40 blocks. There were 56, 54 and 64 deviant tones in each run respectively. The subject then returned on a separate day and repeated the first two runs of the experiment in the MEG (which differed only in the human-controlled stimulus timings).

### Stimulus presentation and onset timing identification.

The experiment was controlled using the Cogent2000 software (<http://www.vislab.ucl.ac.uk/cogent_2000.php>). In both the MRI and MEG scanners, auditory stimuli were triggered using a low-latency audio presentation system (AudioFile Stimulus Processor, Cambridge Research Systems, Rochester, UK) and delivered to the subject using the Ear-Tone Etymotic stereo sound system (Etymotic Research Inc., Illinois, USA). The timing of auditory stimuli and button presses were recorded in the MRI scanner using a Micro 1401 Mk II connected to a computer running the Spike2 software version 6 (Cambridge Electronics Devices, Cambridge, England).

There were four experimental conditions in the paradigm, and the onsets and offset times of each trial needed to be identified precisely for the analyses presented here. These were $(i)$ standard tones in the control block, $(ii)$ deviant tones in the control block, $(iii)$ deviant tones in the respond block and $(iv)$ standard tones in the respond block. For simplicity, the first tone of a new frequency or block was defined as a deviant, and all other tones were defined as standards. The onset of each trial was detected from the Spike2 audio recording, in order to account for any latency in audio presentation. This detection was based on a pre-defined threshold, and the identity of the experimental condition was determined from the auditory cues using a classification method that utilized the dynamic time warping (DTW) algorithm. To clarify this, first we saved two separate audio files for the words ‘control’ and ‘respond’ from the audio files (hereinafter called templates). Next, using the pre-defined threshold, we detected the onset and offset of each word in the timeline of the experiment. These onsets/offsets were employed to detect the timing of each individual word. Each individual word was then compared with the two audio templates using dynamic time warping (DTW) distance. A word in the experiment timeline was identified as control (respond) if its DTW distance to the template audio file of the word ‘control’ (‘respond’) was smaller than ‘respond’ (‘control’).

### MRI data acquisition & preprocessing

A 2D gradient echo planar imaging sequence was used to acquire the functional data in a 3T Prisma scanner (Siemens Healthineers, Erlangen, Germany). A 64 channel coil was used for signal reception and an integrated body coil for transmission. Each volume consisted of 48 transverse slices and was acquired in 3.36s, with ascending slice order. The following parameters were used: voxel size of 3 mm x 3 mm in-plane; slice thickness of 2.5mm, 0.5mm slice separation, field of view of 192 mm × 192 mm, 12% over-sampling in the phase-encoded direction, bandwidth of 2298 Hz/px, echo spacing 0.5ms, echo time of 30 ms; flip angle of 90°. Fat saturation with an excitation of 130 degrees was used prior to each excitation. A fieldmap was also acquired to correct the images for distortions.

A T1-weighted MPRAGE (magnetisation-prepared 3D rapid gradient echo) anatomical image (Mugler and Brookeman, MRM, 1990) was acquired with the following parameters: inversion time was set to 1100 ms; excitation flip angle was 7°; time to echo was 3.34 ms; receiver bandwidth was 200 Hz/pixel; echo spacing was 7.4 ms; and repetition time, 2530 ms. Reconstruction matrix dimensions were 256×256×176, with 1×1×1 mm3 voxel size. Parallel imaging acceleration (Griswold et al., MRM, 2002) was enabled with acceleration factor of 2 and 32 integrated reference lines, giving a scan time of 6:03 minutes.

Functional and structural images were bias-corrected and then pre-processed using the standard pipeline in SPM12 (revision 7265). Functional images were realigned and unwarped using the acquired fieldmaps and the structural image was segmented into constituent tissue types. The functional images were co-registered to the structural, and all images were normalised to MNI space. Explicit smoothing with a 6mm FWHM kernel was applied to the functional images, in order to increase SNR and to satisfy the requirements of multiple comparisons correction using random field theory.

The preprocessed fMRI data were analysed by specifying a single General Linear Model (GLM) for each subject, concatenated over runs. This included regressors for the deviants in the respond condition, deviants in the control condition, cues, physiological regressors derived from breathing / pulse measurements and a constant term for each run. The GLM was estimated and results assessed at a voxel-wise threshold of p < 0.05 family-wise error (FWE) corrected, shown in Figure S1 and Table S1.


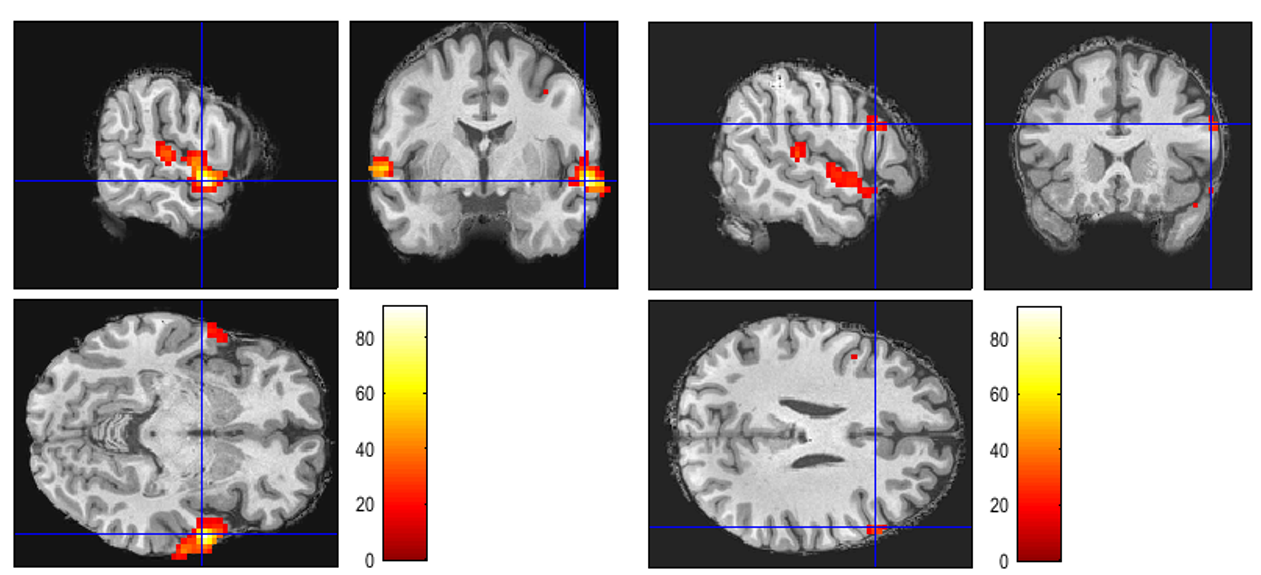


**Figure S1. Functional MRI results.** The panels show the thresholded Statistical Parametric Mapping (SPM) results with sagittal and coronal planes on the top row and the axial plane on the bottom row. **Left**: the crosshair positioned on right planum temporale (rPT), MNI coordinates: [69, -19, 2]. **Right**: the crosshair positioned on right inferior frontal gyrus (rIFG), MNI coordinates: [54, 20, 26].

Regions of interest were identified by positioning spheres of radius 8mm at the coordinate of peak activation of each region (listed in Table S1). Each region’s time series was summarised over significant voxels (p < 0.05 FWE corrected) by taking their first principal component (eigenvariate). Each summary timeseries was high pass filtered, pre-whitened, and corrected for known confounds (the mean and physiological regressors). These time series were used for the haemodynamic modelling, below. An additional GLM was specified including all standards and deviant tones, which provided the timing information needed for the haemodynamic modelling.

### MEG data acquisition & pre-processing

MEG recordings were made using a 275-channel Canadian ThinFilms (CTF) MEG system with superconducting quantum interference device (SQUID)-based axial gradiometers (VSM MedTech, Vancouver, Canada) in a magnetically shielded room. The data collected (included button presses and onsets of audio stimuli) were digitized continuously at a sampling rate of 600 Hz.

The data were first epoched into different segments. Each segment was the time course of one of the experimental conditions; namely a standard tone in a control block, a deviant tone in a control block, a deviant tone in a respond block and a standard tone in a respond block. Each segment was high and low pass filtered in the range of $[0.5 35]$ HZ, respectively. Then we used the conventional averaging method to calculate evoked responses associated with each trial within a range of $[0 400]$ ms for each individual condition. The ensuing evoked responses for different conditions and channels are shown in Figure S2.

Figure S2 Average evoked responses from the MEG data. The left hand side shows traces of evoked responses over all channels associated with standard tones in respond blocks (SR), deviant tones in respond blocks (DR), standard tones in control blocks (SC), and deviant tones in control blocks (DC). The right hand side shows the heat map of changes of brain activity over different channels for SR, DR, SC and DC evoked responses.

## Supplementary table 1: SPM results

| **Contrast** | **Region** | **T-statistic (peak)** | **Coordinates (xyz)** |
| --- | --- | --- | --- |
| Deviants - Standards | Right planum temporale | 13.48 | 69 -19 2 |
| Deviants - Standards | Left planum temporale | 9.49 | -69 -25 8 |
| Deviants - Standards | Right IFG | 8.16 | 54 20 26 |
| Auditory cues | Right HG | 7.47 | 45 -22 8 |
| Auditory cues | Left HG | 8.97 | -42 -28 8 |

* Results were computed at p < 0.05 FWE-corrected, limited to a priori regions of interest based on previous studies.

## Supplementary text: subject briefing

The wording used to brief the subject before scanning was as follows:

*This experiment is all about investigating how we hear sound. In the scanner, you’ll be wearing headphones and holding some buttons which you can press.*

*Sometimes you’ll hear the computer say the word “respond”, and then you’ll hear some beeps. Your task is simply to press a button every time you hear a beep. Try and press it as soon as you can when you hear a beep.*

*At other times, the computer will say the word “control”. Now, you’re in control of the beeps. Every time you press a button, you’ll hear a beep. I’d like you press the button at a similar speed as the beeps you heard the computer making when you were responding. But I’d like you to mix it up a bit – sometimes press the button a bit faster, sometimes press it a bit slower. You are in control.*

*So it’s as simple as that. When the computer says “respond”, press your button when you hear a beep. When the computer says “control”, press your button at your own pace. Keep looking at the cross in the middle of the screen during the task (feel free to blink). Any questions?*

## References

Garrido MI, Friston KJ, Kiebel SJ, Stephan KE, Baldeweg T, Kilner JM. (2008) The functional anatomy of the MMN: a DCM study of the roving paradigm. Neuroimage. 15;42(2):936-44.
